# Supplementary material for: Phenotypic diversity and provenance variation of Cupressus funebris: a case study in the Sichuan Basin, China
Source: PeerJ. 2024 Nov 29;12:e18494. doi: 10.7717/peerj.18494 (PMC11610466; doi:10.7717/peerj.18494)
Supplement: Supplemental Information 2 — Notes: The specific location of the provenance is shown in Figure S1. Among the provenance, BZ represents Bazhong City, GY represents Guangyuan City, NC represents Nanchong City, NJ represents Nanjiang County, and ST represents Santai County. [file peerj-12-18494-s002.docx]

| provenance | Code (count) | Longitude | Latitude | Average Altitude (m) | Average Annual Temperature (◦C) | Average Annual Rainfall (mm) |
| --- | --- | --- | --- | --- | --- | --- |
| BZ | BZ1-BZ33 (33) | 106°40′ E | 32°05′ N | 801 | 17.2 | 1189 |
| GY | GY1-GY30 (30) | 105°40′ E | 32°10′ N | 900 | 16.1 | 900 |
| NC | NC1-NC21 (21) | 106°07′ E | 31°10′ N | 572 | 17.1 | 1000 |
| NJ | NJ1-NJ78 (78) | 106°26′ E | 31°52′ N | 970 | 16.2 | 1200 |
| ST | ST1-ST18 (18) | 104º43′ E | 30º42′ N | 450 | 16.7 | 882 |
