# Supplementary material for: Phenotypic diversity and provenance variation of Cupressus funebris: a case study in the Sichuan Basin, China
Source: PeerJ. 2024 Nov 29;12:e18494. doi: 10.7717/peerj.18494 (PMC11610466; doi:10.7717/peerj.18494)
Supplement: Supplemental Information 3 — Notes: ABA: annual branch angle; BH: branch height; CH: crown height; CH/CW: the ratio of crown height to crown width; COV: cone volume; CSN: cone scales number; CTD: cone transverse diameter; CVD: cone vertical diameter; CW: crown width; DBH: diameter at breast height; H: tree height; H/CW: the ratio of tree height to crown width; H/CH: the ratio of tree height to crown height; HGW: hundred-grain weight; LA: leaf angle; LAB: the length of annual branch; SL: seed length; SW: seed width; V: wood volume. [file peerj-12-18494-s003.docx]

| Triat | Unit | Mean | Max | Min | Range | SD |
| --- | --- | --- | --- | --- | --- | --- |
| H | m | 12.4 | 10 | 15 | 5 | 1.28 |
| DBH | cm | 27.6 | 21 | 35.5 | 14.5 | 3.04 |
| V | m³ | 0.37 | 0.17 | 0.7 | 0.53 | 0.1 |
| CW | m | 6.9 | 4.6 | 9.6 | 5 | 1.09 |
| BH | m | 4.6 | 2 | 9 | 7 | 1.11 |
| CH | m | 7.8 | 4.7 | 11.4 | 6.7 | 1.25 |
| H/CW | - | 1.8 | 1.4 | 3 | 1.6 | 0.29 |
| CH/CW | - | 1.2 | 0.64 | 2.07 | 1.43 | 0.23 |
| H/CH | - | 1.6 | 1.18 | 2.76 | 1.58 | 0.22 |
| LAB | cm | 27.5 | 15.6 | 45.5 | 29.9 | 4.76 |
| ABA | - | 60.8 | 35.1 | 95.4 | 60.3 | 8.64 |
| LA | - | 40.8 | 27.1 | 55.7 | 28.6 | 5.24 |
| CVD | mm | 10.2 | 13.6 | 8 | 5.6 | 1.03 |
| CTD | mm | 10.3 | 13.3 | 8.3 | 5 | 1.03 |
| COV | cm³ | 0.581 | 1.27 | 0.28 | 0.99 | 0.18 |
| CSN | - | 7.7 | 5.9 | 9.9 | 4 | 0.73 |
| SL | mm | 2.62 | 3.48 | 2.05 | 1.43 | 0.26 |
| SW | mm | 2.66 | 3.93 | 1.18 | 2.75 | 0.46 |
| HGW | g | 0.19 | 0.43 | 0.09 | 0.34 | 0.06 |
