# Supplementary material for: Phenotypic diversity and provenance variation of Cupressus funebris: a case study in the Sichuan Basin, China
Source: PeerJ. 2024 Nov 29;12:e18494. doi: 10.7717/peerj.18494 (PMC11610466; doi:10.7717/peerj.18494)
Supplement: Supplemental Information 4 — Notes: MS: mean squares; df: degrees of freedom. *: p < 0.05; **: p < 0.01. ABA: annual branch angle; BH: branch height; CH: crown height; CH/CW: the ratio of crown height to crown width; COV: cone volume; CSN: cone scales number; CTD: cone transverse diameter; CVD: cone vertical diameter; CW: crown width; DBH: diameter at breast height; H: tree height; H/CW: the ratio of tree height to crown width; H/CH: the ratio of tree height to crown height; HGW: hundred-grain weight; LA: leaf angle; LAB: the length of annual branch; SL: seed length; SW: seed width; V: wood volume. [file peerj-12-18494-s004.docx]

| Traits | MS (df) | | | F Value | |
| --- | --- | --- | --- | --- | --- |
|  | Among  Provenances | Within  Provenances | Error | Among  Provenances | Within  Provenances |
| H | 44.831(4) | 1.032(55) | 0.462(120) | 43.43^**^ | 2.23^**^ |
| DBH | 170.682(4) | 7.564(55) | 4.608(120) | 22.57^**^ | 1.64^**^ |
| V | 0.216(4) | 0.006(55) | 0.004(120) | 35.97^**^ | 1.5^*^ |
| CW | 18.224(4) | 1.265(55) | 0.571(120) | 14.4^**^ | 2.22^**^ |
| BH | 17.755(4) | 1.277(55) | 0.660(120) | 13.9^**^ | 1.93^**^ |
| CH | 9.643(4) | 2.309(55) | 0.942(120) | 4.18^**^ | 2.45^**^ |
| H/CW | 1.977(4) | 0.074(55) | 0.023(120) | 26.57^**^ | 3.2^**^ |
| CH/CW | 0.723(4) | 0.071(55) | 0.023(120) | 10.2^**^ | 3.14^**^ |
| H/CH | 0.226(4) | 0.075(55) | 0.033(120) | 2.99^*^ | 2.32^**^ |
| LAB | 241.748(4) | 38.307(55) | 8.221(120) | 6.31^**^ | 4.66^**^ |
| ABA | 434.379(4) | 145.813(55) | 29.948(120) | 2.98^*^ | 4.87^**^ |
| LA | 149.011(4) | 49.652(55) | 13.157(120) | 3^*^ | 3.77^**^ |
| CVD | 11.128(4) | 1.624(55) | 0.468(120) | 6.85^**^ | 3.47^**^ |
| CTD | 9.314(4) | 1.775(55) | 0.446(120) | 5.25^**^ | 3.98^**^ |
| COV | 0.390(4) | 0.051(55) | 0.013(120) | 7.68^**^ | 3.81^**^ |
| CSN | 1.967(4) | 0.854(55) | 0.334(120) | 2.3 | 2.55^**^ |
| SL | 0.727(4) | 0.106(55) | 0.031(120) | 6.85^**^ | 3.47^**^ |
| SW | 1.661(4) | 0.472(55) | 0.08(120) | 3.52^*^ | 5.88^**^ |
| HGW | 0.053(4) | 0.006(55) | 0.001(120) | 9.67^**^ | 3.93^**^ |
