# Supplementary material for: Phenotypic diversity and provenance variation of Cupressus funebris: a case study in the Sichuan Basin, China
Source: PeerJ. 2024 Nov 29;12:e18494. doi: 10.7717/peerj.18494 (PMC11610466; doi:10.7717/peerj.18494)
Supplement: Supplemental Information 5 — Notes: ABA: annual branch angle; BH: branch height; CH: crown height; CH/CW: the ratio of crown height to crown width; COV: cone volume; CSN: cone scales number; CTD: cone transverse diameter; CVD: cone vertical diameter; CW: crown width; DBH: diameter at breast height; H: tree height; H/CW: the ratio of tree height to crown width; H/CH: the ratio of tree height to crown height; HGW: hundred-grain weight; LA: leaf angle; LAB: the length of annual branch; SL: seed length; SW: seed width; V: wood volume. [file peerj-12-18494-s005.docx]

| Trait | Components | | | | | Overall  Score |
| --- | --- | --- | --- | --- | --- | --- |
|  | PCA1 | PCA2 | PCA3 | PCA4 | PCA5 |  |
| H | 0.073 | 0.02 | 0.233 | 0.257 | 0.019 | 0.602 |
| DBH | 0.069 | -0.116 | 0.228 | 0.039 | -0.111 | 0.109 |
| V | 0.086 | -0.078 | 0.269 | 0.113 | -0.089 | 0.301 |
| CW | 0.086 | -0.119 | 0.218 | -0.146 | 0.176 | 0.215 |
| BH | 0.048 | -0.21 | -0.027 | 0.297 | -0.019 | 0.089 |
| CH | 0.032 | 0.207 | 0.262 | -0.001 | 0.036 | 0.536 |
| H/CW | -0.038 | 0.156 | -0.062 | 0.367 | -0.177 | 0.246 |
| CH/CW | -0.041 | 0.277 | 0.035 | 0.158 | -0.105 | 0.324 |
| H/CH | 0.017 | -0.251 | -0.136 | 0.209 | -0.032 | -0.193 |
| LAB | -0.011 | 0.05 | 0.099 | -0.089 | 0.464 | 0.513 |
| ABA | -0.039 | 0.038 | -0.02 | 0.137 | 0.513 | 0.629 |
| LA | 0.006 | 0.032 | -0.043 | 0.241 | 0.424 | 0.66 |
| CVD | 0.149 | 0.056 | -0.102 | -0.021 | 0.013 | 0.095 |
| CTD | 0.146 | 0.061 | -0.092 | -0.042 | -0.051 | 0.022 |
| COV | 0.151 | 0.061 | -0.081 | -0.032 | -0.006 | 0.093 |
| CSN | 0.049 | -0.033 | -0.094 | -0.005 | 0.401 | 0.318 |
| SL | 0.149 | 0.057 | -0.102 | -0.021 | 0.012 | 0.095 |
| SW | 0.117 | 0.071 | -0.067 | 0.066 | -0.032 | 0.155 |
| HGW | 0.152 | 0.058 | -0.074 | -0.022 | -0.006 | 0.108 |
