# Supplementary material for: Phenotypic diversity and provenance variation of Cupressus funebris: a case study in the Sichuan Basin, China
Source: PeerJ. 2024 Nov 29;12:e18494. doi: 10.7717/peerj.18494 (PMC11610466; doi:10.7717/peerj.18494)
Supplement: Supplemental Information 6 — Notes: ABA: annual branch angle; BH: branch height; CH: crown height; CH/CW: the ratio of crown height to crown width; COV: cone volume; CSN: cone scales number; CTD: cone transverse diameter; CVD: cone vertical diameter; CW: crown width; DBH: diameter at breast height; H: tree height; H/CW: the ratio of tree height to crown width; H/CH: the ratio of tree height to crown height; HGW: hundred-grain weight; LA: leaf angle; LAB: the length of annual branch; SL: seed length; SW: seed width; V: wood volume. [file peerj-12-18494-s006.docx]

| Trait | Groups | | | | |
| --- | --- | --- | --- | --- | --- |
|  | Ⅰ | Ⅱ | Ⅲ | Ⅳ | Average |
| H | 12.5 | 12.8 | 12.0 | 12.6 | 12.4 |
| DBH | 27.1 | 26.9 | 26.3 | 29.1 | 27.3 |
| V | 0.358 | 0.355 | 0.321 | 0.407 | 0.360 |
| CW | 6.9 | 6.1 | 6.6 | 7.2 | 6.7 |
| BH | 4.6 | 4.8 | 4.2 | 4.9 | 4.6 |
| CH | 7.9 | 7.9 | 7.8 | 7.7 | 7.8 |
| H/CW | 1.8 | 2.1 | 1.8 | 1.8 | 1.9 |
| CH/CW | 1.2 | 1.3 | 1.2 | 1.1 | 1.2 |
| H/CH | 1.6 | 1.6 | 1.6 | 1.7 | 1.6 |
| LAB | 28.2 | 28.8 | 30.0 | 25.1 | 28.0 |
| ABA | 53.2 | 75.9 | 64.8 | 62.2 | 64.0 |
| LA | 39.7 | 43.8 | 37.0 | 42.8 | 40.8 |
| CVD | 10.35 | 10.02 | 9.93 | 10.30 | 10.15 |
| CTD | 10.43 | 9.96 | 9.97 | 10.39 | 10.19 |
| COV | 0.601 | 0.541 | 0.526 | 0.600 | 0.567 |
| CSN | 7.7 | 7.5 | 7.7 | 7.7 | 7.6 |
| SL | 2.65 | 2.56 | 2.54 | 2.64 | 2.60 |
| SW | 2.69 | 2.53 | 2.48 | 2.76 | 2.62 |
| HGW | 0.197 | 0.179 | 0.170 | 0.196 | 0.186 |
