# Supplementary material for: Phenotypic diversity and provenance variation of Cupressus funebris: a case study in the Sichuan Basin, China
Source: PeerJ. 2024 Nov 29;12:e18494. doi: 10.7717/peerj.18494 (PMC11610466; doi:10.7717/peerj.18494)
Supplement: Supplemental Information 7 — Notes: ABA: annual branch angle; BH: branch height; CH: crown height; CH/CW: the ratio of crown height to crown width; COV: cone volume; CSN: cone scales number; CTD: cone transverse diameter; CVD: cone vertical diameter; CW: crown width; DBH: diameter at breast height; H: tree height; H/CW: the ratio of tree height to crown width; H/CH: the ratio of tree height to crown height; HGW: hundred-grain weight; LA: leaf angle; LAB: the length of annual branch; SL: seed length; SW: seed width; V: wood volume. [file peerj-12-18494-s007.docx]

| Traits | BZ | GY | NC | NJ | ST | All samples |
| --- | --- | --- | --- | --- | --- | --- |
| H | 7.1 | 5.9 | 5.1 | 7.1 | 4.8 | 10.3 |
| DBH | 9.6 | 6.0 | 9.2 | 8.6 | 8.9 | 11 |
| V | 24.3 | 13.3 | 17.9 | 19.3 | 15.9 | 26.3 |
| CW | 9.6 | 13.3 | 15.6 | 14.3 | 8.5 | 15.8 |
| BH | 18.3 | 21.2 | 19.1 | 20.5 | 17.4 | 24 |
| CH | 11.6 | 15.1 | 13.1 | 17.5 | 10.2 | 16 |
| H/CW | 6.6 | 13.3 | 13.7 | 10.1 | 4.6 | 15.9 |
| CH/CW | 11.2 | 22.5 | 20.7 | 15.6 | 10.7 | 20.1 |
| H/CH | 9.4 | 15.2 | 16.7 | 13.0 | 9.9 | 14 |
| LAB | 11.8 | 14.3 | 13.2 | 17.3 | 16.7 | 17.3 |
| ABA | 8.6 | 8.9 | 21.6 | 11.5 | 20.2 | 14.2 |
| LA | 15.2 | 12.5 | 6.8 | 12.2 | 10.8 | 12.8 |
| CVD | 6.4 | 10.6 | 9.0 | 8.6 | 10.4 | 10.1 |
| CTD | 7.8 | 10.9 | 8.1 | 8.3 | 11.1 | 10 |
| COV | 20.5 | 31.8 | 25.0 | 25.3 | 31.4 | 31.3 |
| CSN | 7.7 | 9.5 | 9.3 | 9.5 | 9.8 | 10.2 |
| SL | 6.3 | 10.6 | 9.0 | 8.6 | 10.5 | 10.1 |
| SW | 18.0 | 27.3 | 5.8 | 15.6 | 10.8 | 18.3 |
| HGW | 20.7 | 32.2 | 24.9 | 25.7 | 31.0 | 32.6 |
| Average | 12.1 | 15.5 | 13.9 | 14.2 | 13.3 | 16.9 |
