# Supplementary material for: Phenotypic diversity and provenance variation of Cupressus funebris: a case study in the Sichuan Basin, China
Source: PeerJ. 2024 Nov 29;12:e18494. doi: 10.7717/peerj.18494 (PMC11610466; doi:10.7717/peerj.18494)
Supplement: Supplemental Information 8 — Notes: ABA: annual branch angle; BH: branch height; CH: crown height; CH/CW: the ratio of crown height to crown width; COV: cone volume; CSN: cone scales number; CTD: cone transverse diameter; CVD: cone vertical diameter; CW: crown width; DBH: diameter at breast height; H: tree height; H/CW: the ratio of tree height to crown width; H/CH: the ratio of tree height to crown height; HGW: hundred-grain weight; LA: leaf angle; LAB: the length of annual branch; SL: seed length; SW: seed width; V: wood volume. [file peerj-12-18494-s008.docx]

| Traits | BZ | GY | NC | NJ | ST | All samples |
| --- | --- | --- | --- | --- | --- | --- |
| H | 1.08 | 1.03 | 1.04 | 1.36 | 0.98 | 1.65 |
| DBH | 1.95 | 1.93 | 1.86 | 2.06 | 1.88 | 2.06 |
| V | 1.57 | 2.01 | 1.72 | 1.99 | 1.77 | 1.99 |
| CW | 1.78 | 1.90 | 1.70 | 2.00 | 1.92 | 2.08 |
| BH | 1.74 | 1.91 | 1.59 | 2.01 | 1.83 | 2.04 |
| CH | 1.99 | 2.03 | 1.82 | 2.01 | 1.75 | 2.07 |
| H/CW | 1.85 | 1.68 | 1.82 | 1.90 | 1.83 | 1.74 |
| CH/CW | 1.96 | 1.82 | 1.57 | 2.04 | 1.81 | 1.99 |
| H/CH | 1.81 | 1.76 | 1.41 | 2.08 | 1.96 | 1.97 |
| LAB | 2.02 | 1.86 | 1.82 | 1.86 | 1.77 | 2.02 |
| ABA | 1.93 | 1.90 | 1.83 | 2.01 | 1.90 | 2 |
| LA | 1.95 | 2.14 | 1.83 | 2.05 | 1.83 | 2.08 |
| CVD | 2.06 | 1.84 | 1.92 | 1.98 | 1.88 | 2.01 |
| CTD | 1.99 | 1.94 | 1.71 | 2.05 | 1.71 | 2.02 |
| COV | 1.88 | 1.75 | 1.93 | 1.95 | 1.83 | 1.89 |
| CSN | 1.88 | 1.63 | 1.50 | 2.01 | 1.77 | 2.03 |
| SL | 1.59 | 1.84 | 1.92 | 1.98 | 1.88 | 2.01 |
| SW | 1.64 | 1.50 | 1.45 | 2.01 | 1.71 | 1.93 |
| HGW | 1.59 | 1.75 | 1.93 | 1.95 | 1.83 | 1.87 |
| Average | 1.80 | 1.80 | 1.70 | 1.96 | 1.78 | 1.97 |
