# Supplementary material for: Phenotypic diversity and provenance variation of Cupressus funebris: a case study in the Sichuan Basin, China
Source: PeerJ. 2024 Nov 29;12:e18494. doi: 10.7717/peerj.18494 (PMC11610466; doi:10.7717/peerj.18494)
Supplement: Supplemental Information 9 — Notes: Family names are based on place names. BZ represents Bazhong City, and Pingchang is located in Bazhong City; GY represents Guangyuan City, and Cangxi, Guangwei, and Guangzhong are all located in Guangyuan City; NC represents Nanchong City, and Langzhong and Nanbu are both located in Nanchong City; NJ represents Nanjiang County, and ST represents Santai County. [file peerj-12-18494-s009.docx]

| Provenance | Family name | Provenance | Family name | Provenance | Family name |
| --- | --- | --- | --- | --- | --- |
| BZ | Bazhong07 | GY | Guangzhong09 | NJ | Nanjiang46 |
| BZ | Bazhong12 | NC | Langzhong02 | NJ | Nanjiang50 |
| BZ | Bazhong37 | NC | Langzhong03 | NJ | Nanjiang53 |
| BZ | Bazhong38 | NC | Langzhong04 | NJ | Nanjiang54 |
| BZ | Bazhong42 | NC | Nanbu05 | NJ | Nanjiang56 |
| BZ | Bazhong46 | NC | Nanbu06 | NJ | Nanjiang57 |
| BZ | Pingchang01 | NC | Nanbu08 | NJ | Nanjiang60 |
| BZ | Pingchang03 | NC | Nanbu09 | NJ | Nanjiang62 |
| BZ | Pingchang05 | NJ | Nanjiang11 | NJ | Nanjiang64 |
| BZ | Pingchang30 | NJ | Nanjiang12 | NJ | Nanjiang65 |
| BZ | Pingchang31 | NJ | Nanjiang29 | NJ | Nanjiang66 |
| GY | Cangxi07 | NJ | Nanjiang32 | NJ | Nanjiang67 |
| GY | Cangxi09 | NJ | Nanjiang33 | NJ | Nanjiang68 |
| GY | Guangwei06 | NJ | Nanjiang37 | NJ | Nanjiang70 |
| GY | Guangwei07 | NJ | Nanjiang38 | ST | Santai05 |
| GY | Guangwei08 | NJ | Nanjiang40 | ST | Santai09 |
| GY | Guangwei10 | NJ | Nanjiang41 | ST | Santai13 |
| GY | Guangzhong02 | NJ | Nanjiang42 | ST | Santai19 |
| GY | Guangzhong03 | NJ | Nanjiang44 | ST | Santai21 |
| GY | Guangzhong07 | NJ | Nanjiang45 | ST | Santai22 |
