# Supplementary material for: Phenotypic diversity and provenance variation of Cupressus funebris: a case study in the Sichuan Basin, China
Source: PeerJ. 2024 Nov 29;12:e18494. doi: 10.7717/peerj.18494 (PMC11610466; doi:10.7717/peerj.18494)
Supplement: Supplemental Information 10 — Notes: ABA: annual branch angle; BH: branch height; CH: crown height; CH/CW: the ratio of crown height to crown width; COV: cone volume; CSN: cone scales number; CTD: cone transverse diameter; CVD: cone vertical diameter; CW: crown width; DBH: diameter at breast height; H: tree height; H/CW: the ratio of tree height to crown width; H/CH: the ratio of tree height to crown height; HGW: hundred-grain weight; LA: leaf angle; LAB: the length of annual branch; SL: seed length; SW: seed width; V: wood volume. * ：p < 0.05; **：p < 0.01. [file peerj-12-18494-s010.docx]

| Traits | MS (df) | | F Value |
| --- | --- | --- | --- |
|  | Family | Error |  |
| H | 1.05(10) | 0.42(22) | 2.49* |
| DBH | 6.00(10) | 5.37(22) | 1.12 |
| V | 0.01(10) | 0.004(22) | 1.36 |
| CW | 0.40(10) | 0.36(22) | 1.09 |
| BH | 0.57(10) | 0.36(22) | 1.6 |
| CH | 1.36(10) | 0.48(22) | 2.83* |
| H/CW | 0.01(10) | 0.01(22) | 0.85 |
| CH/CW | 0.03(10) | 0.01(22) | 2.17 |
| H/CH | 0.03(10) | 0.01(22) | 2.69* |
| LAB | 19.04(10) | 7.57(22) | 2.52* |
| ABA | 20.65(10) | 28.36(22) | 0.73 |
| LA | 95.62(10) | 12.06(22) | 7.93** |
| CVD | 0.82(10) | 0.22(22) | 3.66** |
| CTD | 1.17(10) | 0.37(22) | 3.14* |
| COV | 0.02(10) | 0.007(22) | 3.33** |
| CSN | 0.50(10) | 0.30(22) | 1.64 |
| SL | 0.06(10) | 0.01(22) | 3.81** |
| SW | 0.42(10) | 0.12(22) | 3.5** |
| HGW | 0.003(10) | 0.001(22) | 3.36** |
