# Supplementary material for: Phenotypic diversity and provenance variation of Cupressus funebris: a case study in the Sichuan Basin, China
Source: PeerJ. 2024 Nov 29;12:e18494. doi: 10.7717/peerj.18494 (PMC11610466; doi:10.7717/peerj.18494)
Supplement: Supplemental Information 11 — Notes: ABA: annual branch angle; BH: branch height; CH: crown height; CH/CW: the ratio of crown height to crown width; COV: cone volume; CSN: cone scales number; CTD: cone transverse diameter; CVD: cone vertical diameter; CW: crown width; DBH: diameter at breast height; H: tree height; H/CW: the ratio of tree height to crown width; H/CH: the ratio of tree height to crown height; HGW: hundred-grain weight; LA: leaf angle; LAB: the length of annual branch; SL: seed length; SW: seed width; V: wood volume. *：p < 0.05; **：p < 0.01. [file peerj-12-18494-s011.docx]

| Traits | MS (df) | | F Value |
| --- | --- | --- | --- |
|  | Family | Error |  |
| H | 0.82(9) | 0.37(20) | 2.23 |
| DBH | 2.88(9) | 2.81(20) | 1.03 |
| V | 0.002(9) | 0.002(20) | 0.86 |
| CW | 2.08(9) | 0.37(20) | 5.6** |
| BH | 2.11(9) | 0.59(20) | 3.55** |
| CH | 2.46(9) | 0.62(20) | 3.99** |
| H/CW | 0.10(9) | 0.03(20) | 3.69** |
| CH/CW | 0.11(9) | 0.03(20) | 3.58** |
| H/CH | 0.13(9) | 0.04(20) | 3.48** |
| LAB | 41.3(9) | 11.49(20) | 3.6** |
| ABA | 63.44(9) | 20.76(20) | 3.06* |
| LA | 75.85(9) | 5.50(20) | 13.78** |
| CVD | 3.22(9) | 0.18(20) | 17.82** |
| CTD | 3.49(9) | 0.19(20) | 18.49** |
| COV | 0.09(9) | 0.004(20) | 23.87** |
| CSN | 1.63(9) | 0.10(20) | 15.61** |
| SL | 0.21(9) | 0.01(20) | 17.82** |
| SW | 1.35(9) | 0.08(20) | 17.49** |
| HGW | 0.009(9) | 0.0003(20) | 23.87** |
