# Supplementary material for: Phenotypic diversity and provenance variation of Cupressus funebris: a case study in the Sichuan Basin, China
Source: PeerJ. 2024 Nov 29;12:e18494. doi: 10.7717/peerj.18494 (PMC11610466; doi:10.7717/peerj.18494)
Supplement: Supplemental Information 12 — Notes: ABA: annual branch angle; BH: branch height; CH: crown height; CH/CW: the ratio of crown height to crown width; COV: cone volume; CSN: cone scales number; CTD: cone transverse diameter; CVD: cone vertical diameter; CW: crown width; DBH: diameter at breast height; H: tree height; H/CW: the ratio of tree height to crown width; H/CH: the ratio of tree height to crown height; HGW: hundred-grain weight; LA: leaf angle; LAB: the length of annual branch; SL: seed length; SW: seed width; V: wood volume. *：p < 0.05; **：p < 0.01. [file peerj-12-18494-s012.docx]

| Traits | MS (df) | | F Value |
| --- | --- | --- | --- |
|  | Family | Error |  |
| H | 1.22(6) | 0.23(14) | 5.35** |
| DBH | 11.79(6) | 3.15(14) | 3.74* |
| V | 0.009(6) | 0.002(14) | 3.67* |
| CW | 2.45(6) | 0.20(14) | 12.42** |
| BH | 2.28(6) | 0.63(14) | 3.6* |
| CH | 2.37(6) | 0.81(14) | 2.94* |
| H/CW | 0.29(6) | 0.03(14) | 9.28** |
| CH/CW | 0.22(6) | 0.04(14) | 5.55** |
| H/CH | 0.13(6) | 0.06(14) | 2.31 |
| LAB | 22.22(6) | 7.50(14) | 2.96* |
| ABA | 579.48(6) | 37.59(14) | 15.42** |
| LA | 21.91(6) | 4.02(14) | 5.45** |
| CVD | 2.43(6) | 0.16(14) | 15.07** |
| CTD | 1.99(6) | 0.12(14) | 16.6** |
| COV | 0.06(6) | 0.003(14) | 17.32** |
| CSN | 1.60(6) | 0.05(14) | 31.97** |
| SL | 0.16(6) | 0.01(14) | 15.07** |
| SW | 0.09(6) | 0.001(14) | 71.83** |
| HGW | 0.007(6) | 0.0004(14) | 17.32** |
