# Supplementary material for: Phenotypic diversity and provenance variation of Cupressus funebris: a case study in the Sichuan Basin, China
Source: PeerJ. 2024 Nov 29;12:e18494. doi: 10.7717/peerj.18494 (PMC11610466; doi:10.7717/peerj.18494)
Supplement: Supplemental Information 13 — Notes: ABA: annual branch angle; BH: branch height; CH: crown height; CH/CW: the ratio of crown height to crown width; COV: cone volume; CSN: cone scales number; CTD: cone transverse diameter; CVD: cone vertical diameter; CW: crown width; DBH: diameter at breast height; H: tree height; H/CW: the ratio of tree height to crown width; H/CH: the ratio of tree height to crown height; HGW: hundred-grain weight; LA: leaf angle; LAB: the length of annual branch; SL: seed length; SW: seed width; V: wood volume. *：p < 0.05; **：p < 0.01. [file peerj-12-18494-s013.docx]

| Traits | MS (df) | | F Value |
| --- | --- | --- | --- |
|  | Family | Error |  |
| H | 1.12(25) | 0.57(52) | 2.08* |
| DBH | 8.91(25) | 4.49(52) | 1.99* |
| V | 0.007(25) | 0.004(52) | 1.6 |
| CW | 1.22(25) | 0.83(52) | 1.46 |
| BH | 1.13(25) | 0.74(52) | 1.51 |
| CH | 3.01(25) | 1.30(52) | 2.33** |
| H/CW | 0.05(25) | 0.03(52) | 2.05* |
| CH/CW | 0.05(25) | 0.02(52) | 2.47* |
| H/CH | 0.07(25) | 0.03(52) | 2.18** |
| LAB | 42.94(25) | 8.39(52) | 5.12** |
| ABA | 111.5(25) | 15.46(52) | 7.21** |
| LA | 36.28(25) | 16.73(52) | 2.17** |
| CVD | 1.02(25) | 0.61(52) | 1.67 |
| CTD | 1.11(25) | 0.51(52) | 2.18** |
| COV | 0.03(25) | 0.01(52) | 2* |
| CSN | 0.54(25) | 0.49(52) | 1.1 |
| SL | 0.07(25) | 0.04(52) | 1.67 |
| SW | 0.32(25) | 0.08(52) | 3.85** |
| HGW | 0.003(25) | 0.002(52) | 2* |
