# Supplementary material for: Phenotypic diversity and provenance variation of Cupressus funebris: a case study in the Sichuan Basin, China
Source: PeerJ. 2024 Nov 29;12:e18494. doi: 10.7717/peerj.18494 (PMC11610466; doi:10.7717/peerj.18494)
Supplement: Supplemental Information 14 — Notes: ABA: annual branch angle; BH: branch height; CH: crown height; CH/CW: the ratio of crown height to crown width; COV: cone volume; CSN: cone scales number; CTD: cone transverse diameter; CVD: cone vertical diameter; CW: crown width; DBH: diameter at breast height; H: tree height; H/CW: the ratio of tree height to crown width; H/CH: the ratio of tree height to crown height; HGW: hundred-grain weight; LA: leaf angle; LAB: the length of annual branch; SL: seed length; SW: seed width; V: wood volume. *：p < 0.05; **：p < 0.01. [file peerj-12-18494-s014.docx]

| Traits | MS (df) | | F Value |
| --- | --- | --- | --- |
|  | Family | Error |  |
| H | 0.36(5) | 0.50(12) | 0.71 |
| DBH | 7.19(5) | 8.35(12) | 0.86 |
| V | 0.01(5) | 0.01(12) | 0.89 |
| CW | 0.34(5) | 0.59(12) | 0.59 |
| BH | 0.73(5) | 0.99(12) | 0.74 |
| CH | 0.33(5) | 0.95(12) | 0.35 |
| H/CW | 0.003(5) | 0.006(12) | 0.45 |
| CH/CW | 0.01(5) | 0.01(12) | 0.63 |
| H/CH | 0.02(5) | 0.03(12) | 0.54 |
| LAB | 67.55(5) | 4.04(12) | 16.72** |
| ABA | 195.50(5) | 102.10(12) | 1.92 |
| LA | 10.71(5) | 23.09(12) | 0.46 |
| CVD | 2.37(5) | 1.13(12) | 2.1 |
| CTD | 2.95(5) | 1.13(12) | 2.62 |
| COV | 0.13(5) | 0.05(12) | 2.83 |
| CSN | 1.04(5) | 0.40(12) | 2.59 |
| SL | 0.15(5) | 0.07(12) | 2.1 |
| SW | 0.19(5) | 0.09(12) | 2.19 |
| HGW | 0.02(5) | 0.005(12) | 2.82 |
