# Supplementary figures and images for: Phenotypic diversity and provenance variation of Cupressus funebris: a case study in the Sichuan Basin, China
Source: PeerJ. 2024 Nov 29;12:e18494. doi: 10.7717/peerj.18494 (PMC11610466; doi:10.7717/peerj.18494)

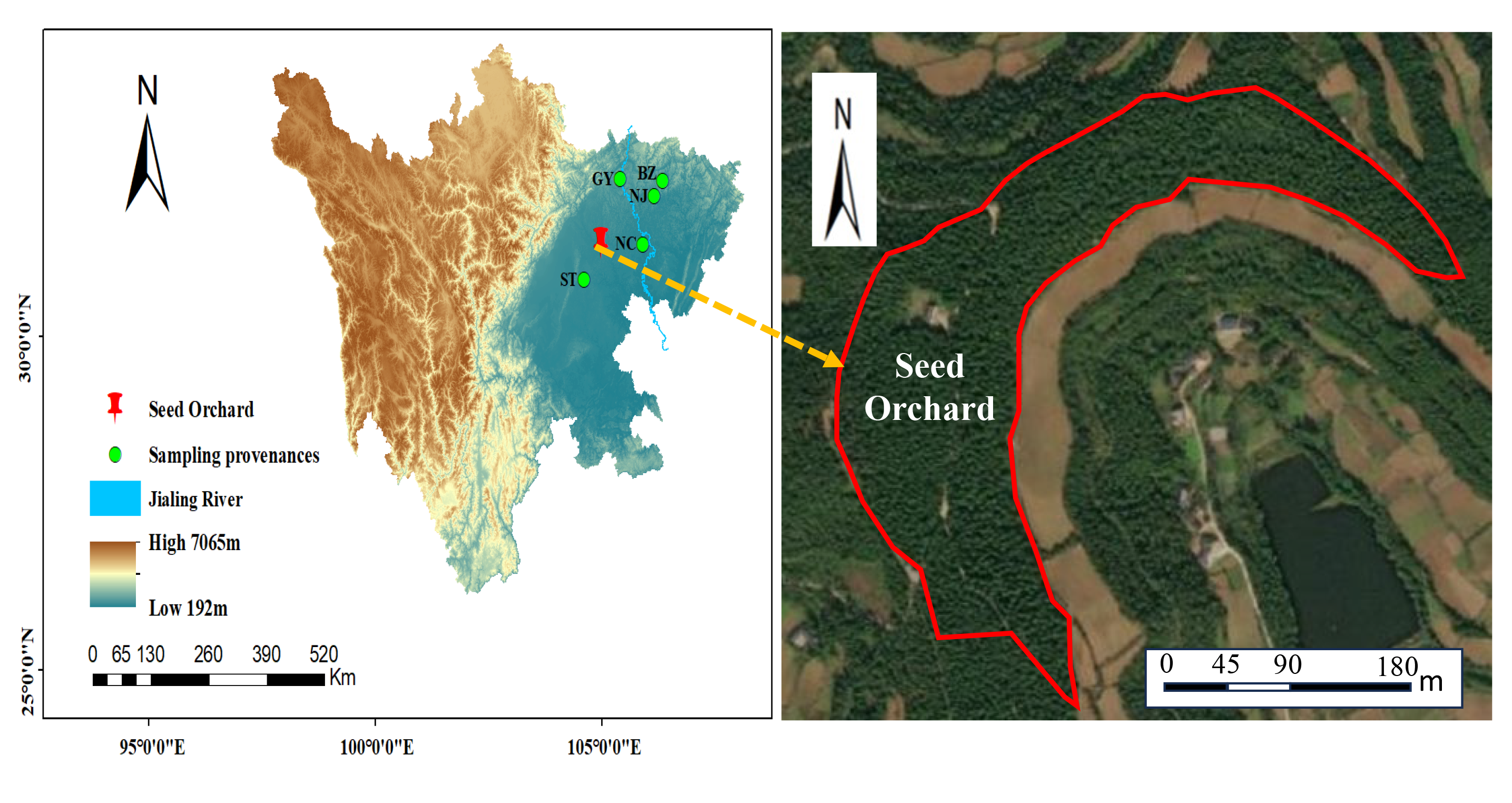

Supplement: Supplemental Information 15 — BZ represents the Bazhong provenance; GY represents the Guangyuan provenance; NC represents the Nanchong provenance; NJ represents the Nanjiang provenance; ST represents the Santai provenance. [file peerj-12-18494-s015.png]

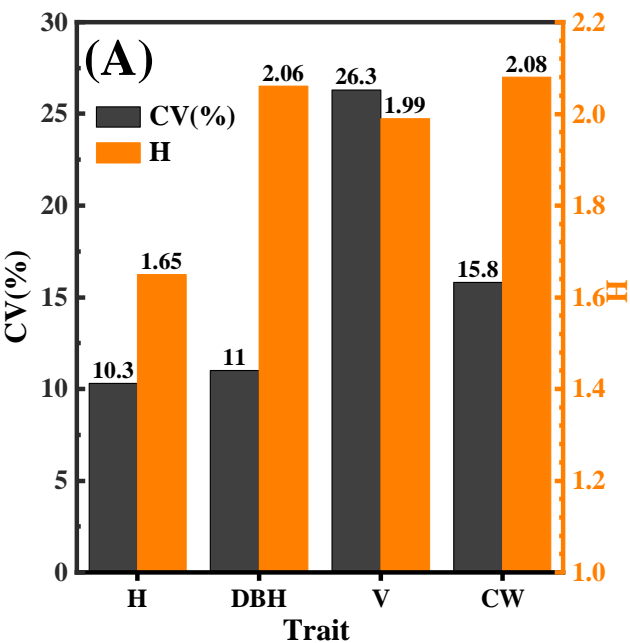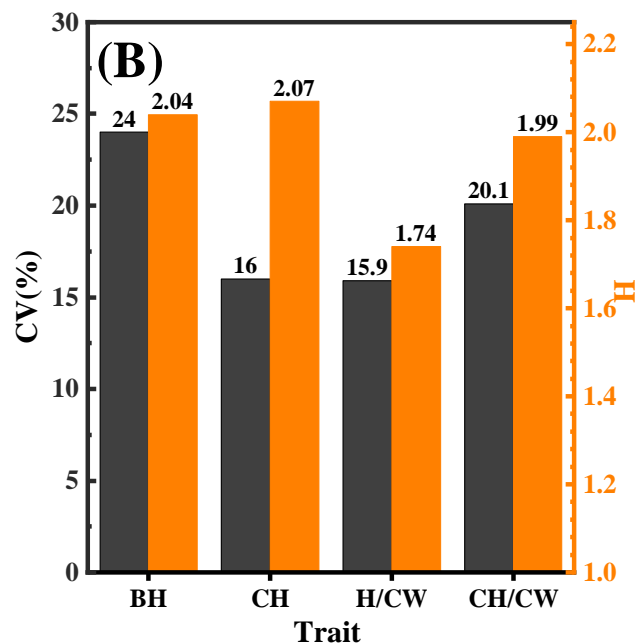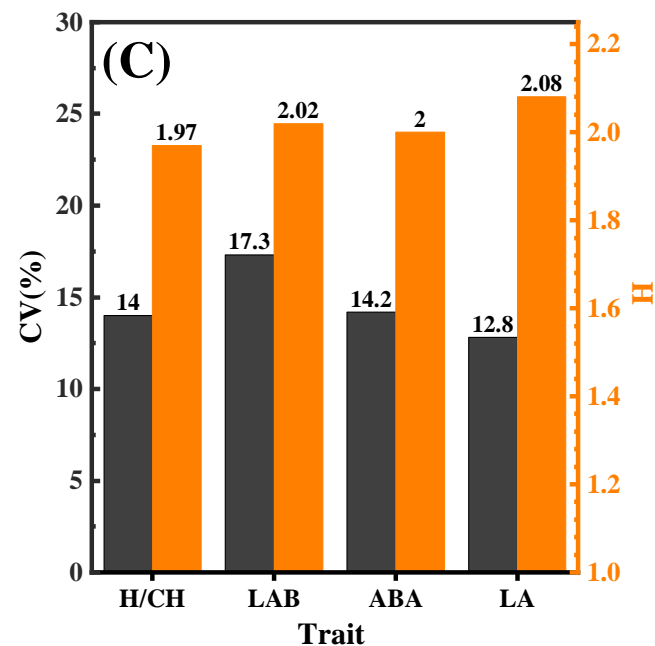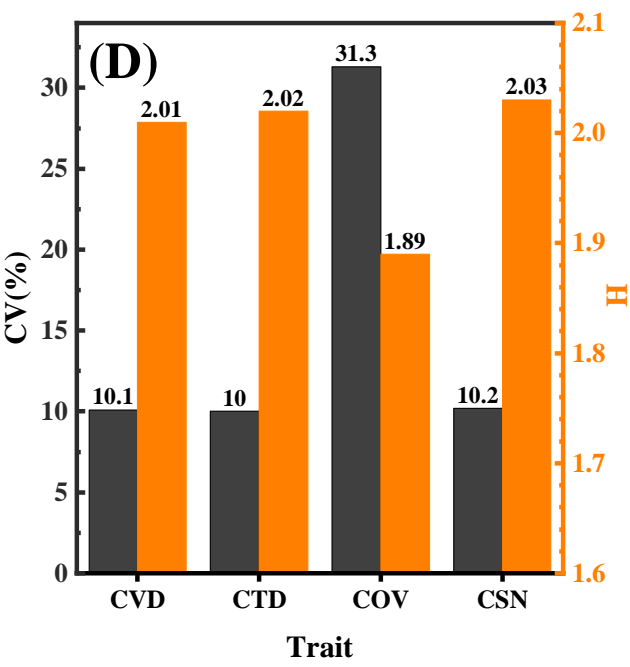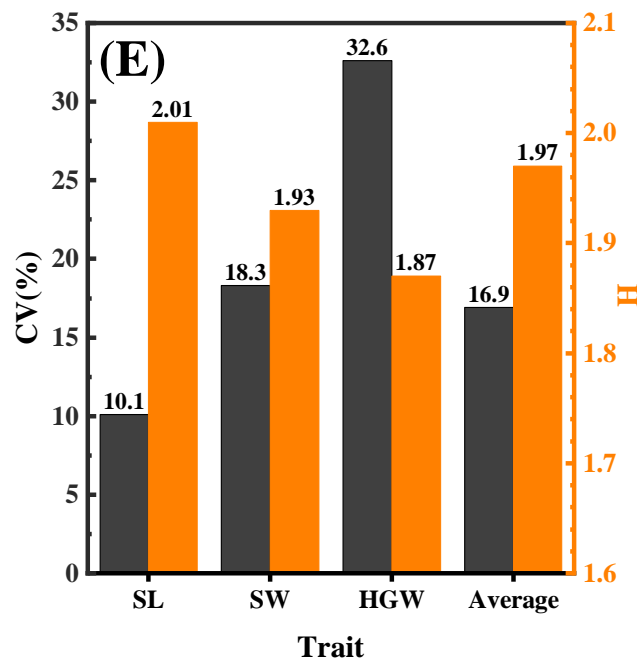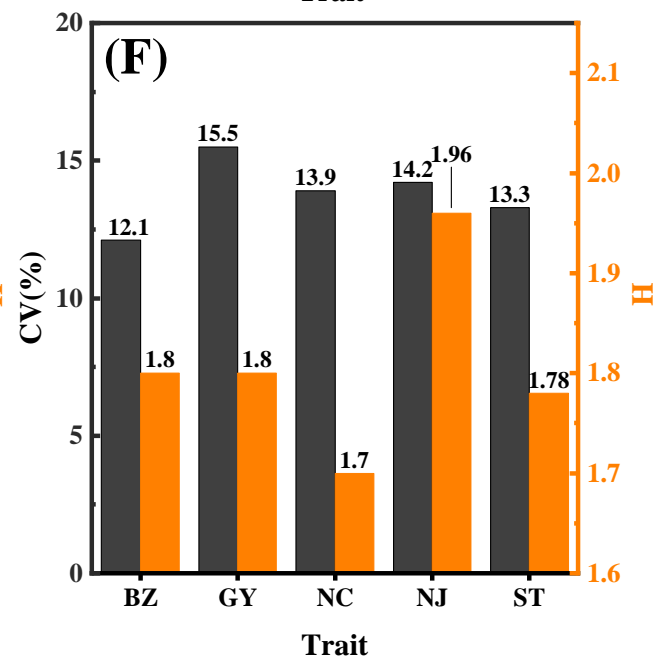

Supplement: Supplemental Information 16 — The numbers above the bar chart represent the coefficient of variation values and the genetic diversity index, respectively. ABA: annual branch angle; BH: branch height; CH: crown height; CH/CW: the ratio of crown height to crown width; COV: cone volume; CSN: cone scales number; CTD: cone transverse diameter; CVD: cone vertical diameter; CW: crown width; DBH: diameter at breast height; H: tree height; H/CW: the ratio of tree height to crown width; H/CH: the ratio of tree height to crown height; HGW: hundred-grain weight; LA: leaf angle; LAB: the length of annual branch; SL: seed length; SW: seed width; V: wood volume. [file peerj-12-18494-s016.pdf]

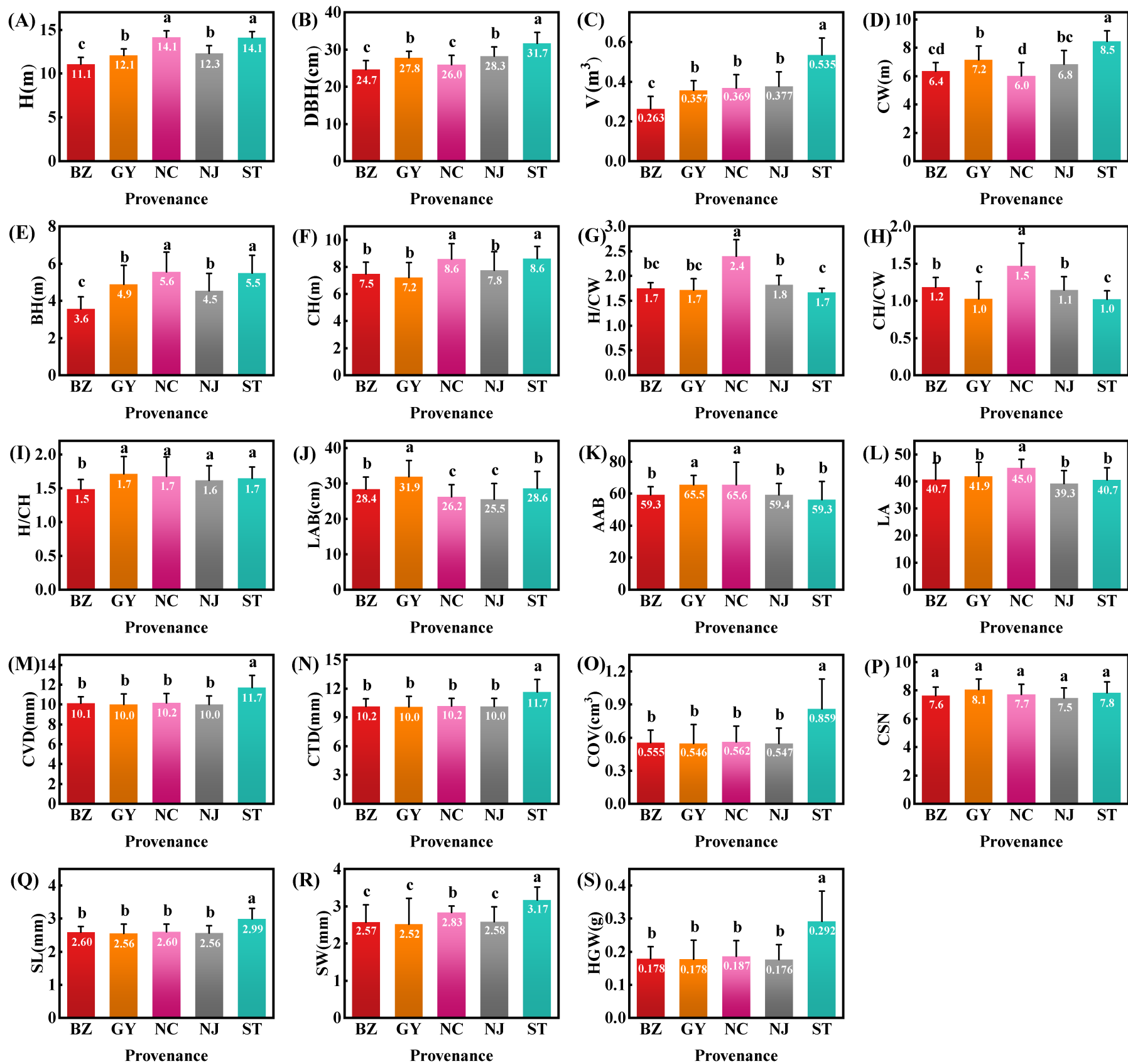

Supplement: Supplemental Information 17 — ABA: annual branch angle; BH: branch height; CH: crown height; CH/CW: the ratio of crown height to crown width; COV: cone volume; CSN: cone scales number; CTD: cone transverse diameter; CVD: cone vertical diameter; CW: crown width; DBH: diameter at breast height; H: tree height; H/CW: the ratio of tree height to crown width; H/CH: the ratio of tree height to crown height; HGW: hundred-grain weight; LA: leaf angle; LAB: the length of annual branch; SL: seed length; SW: seed width; V: wood volume. The numbers in the bar chart represent the average values of the traits. [file peerj-12-18494-s017.pdf]

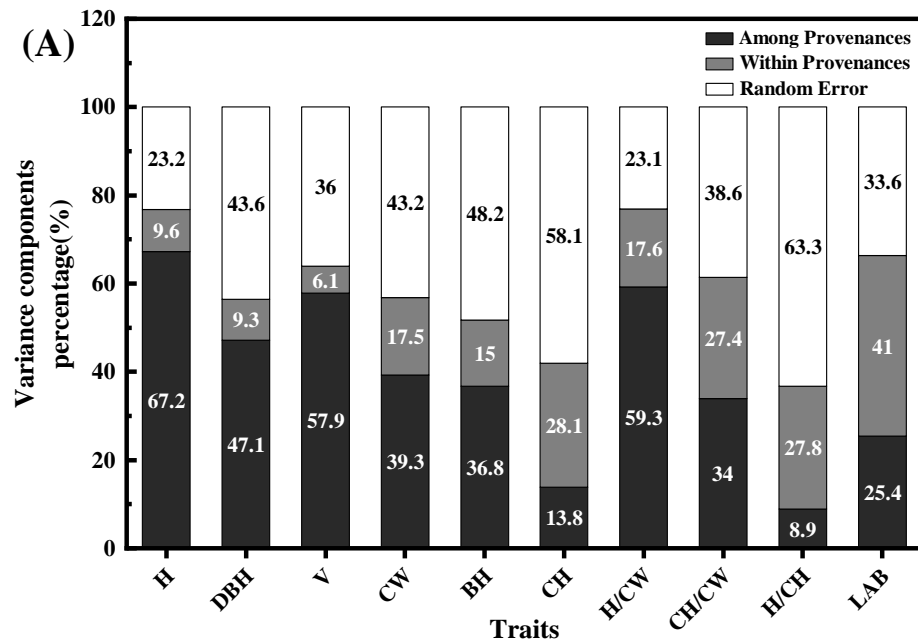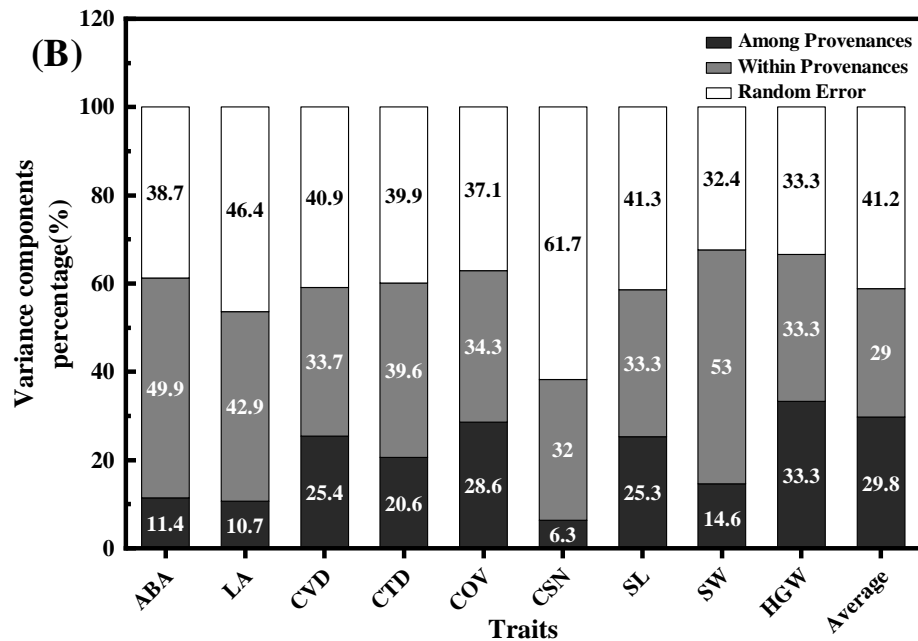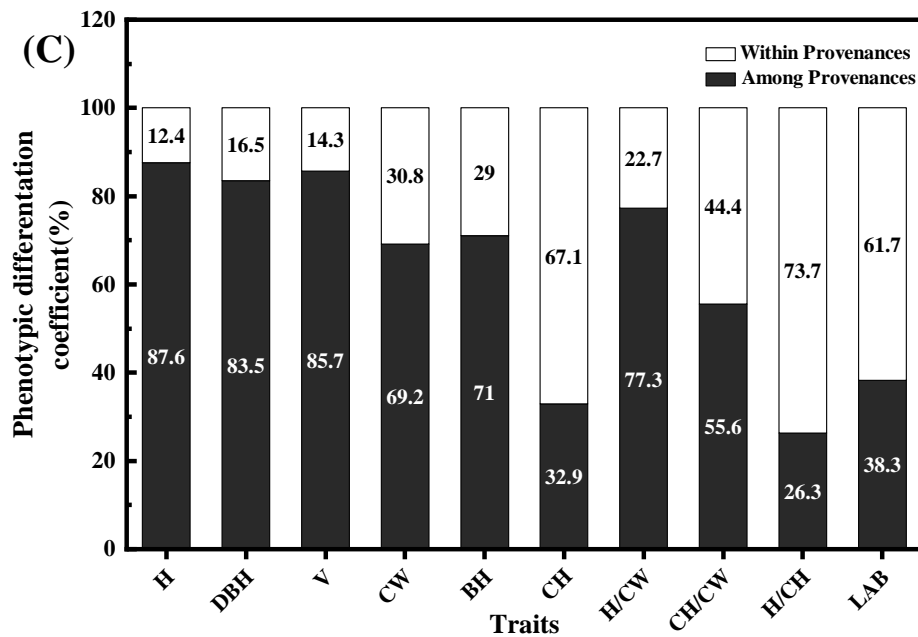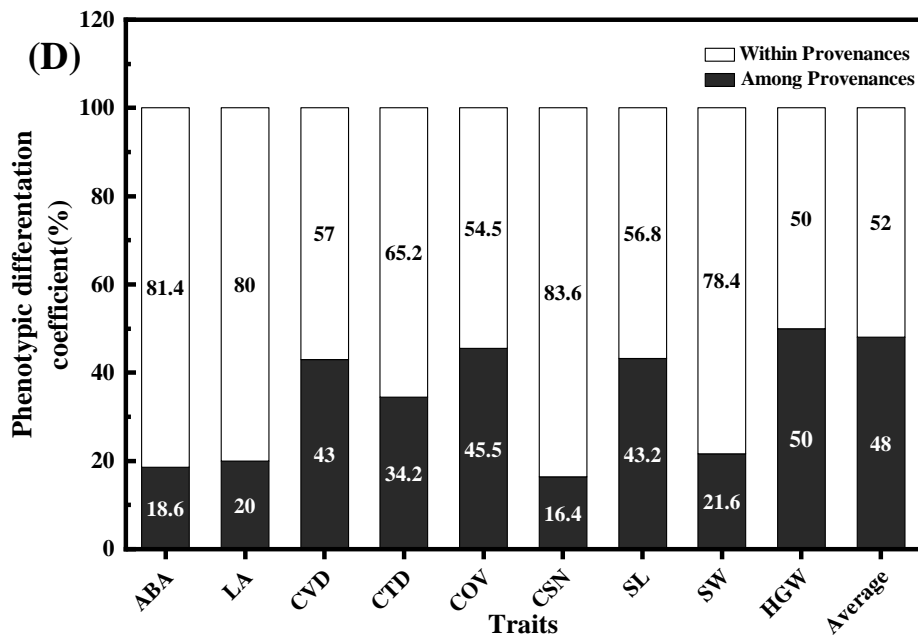

Supplement: Supplemental Information 18 — Variance components percentage of 19 traits (A and B); phenotypic differentiation Coefficient (Vst) among and within provenances (C and D). The information corresponding to the abbreviations can be found in Figure S2. [file peerj-12-18494-s018.pdf]

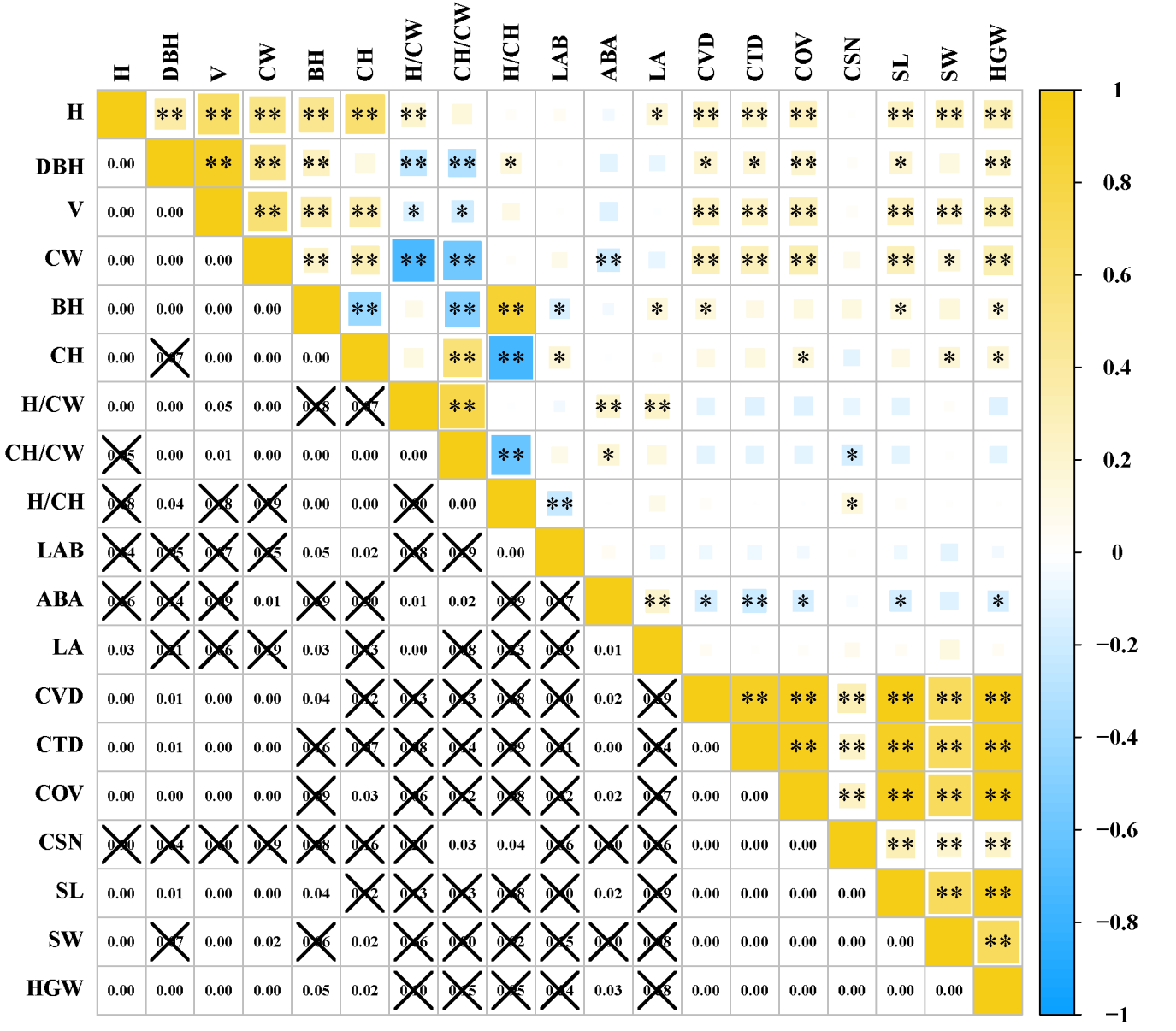

Supplement: Supplemental Information 19 — The upper triangular region of the figure represents the coefficient of association, with darker colors indicating stronger correlations. Significance levels are marked as follows: *: p < 0.05, indicating statistical significance; **: p < 0.01, indicating a higher level of statistical significance. The lower triangular part of the figure indicates the corresponding p-values, and each X mark represents the absence of a significant correlation. The information corresponding to the abbreviations can be found in Figure S2. [file peerj-12-18494-s019.pdf]

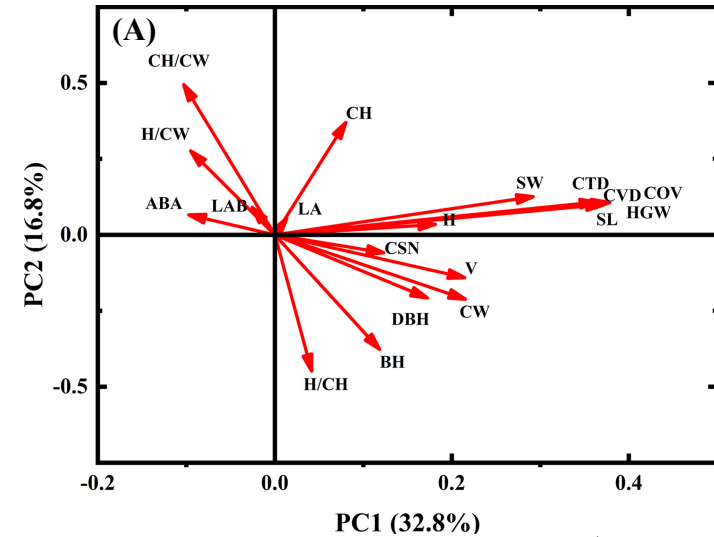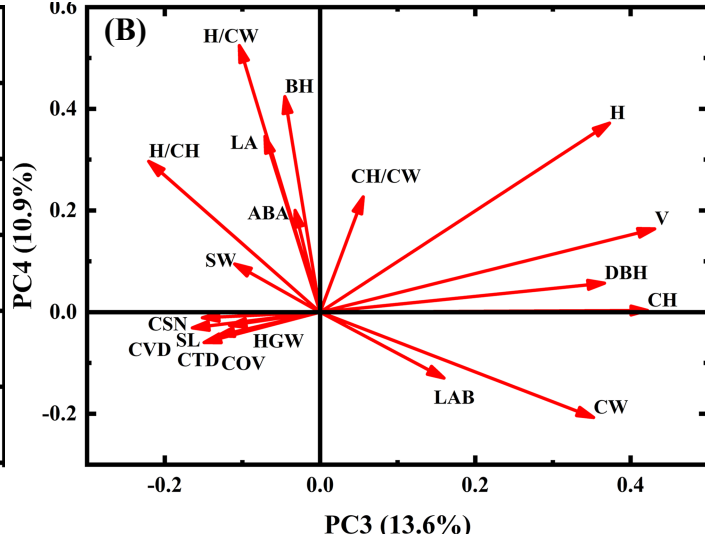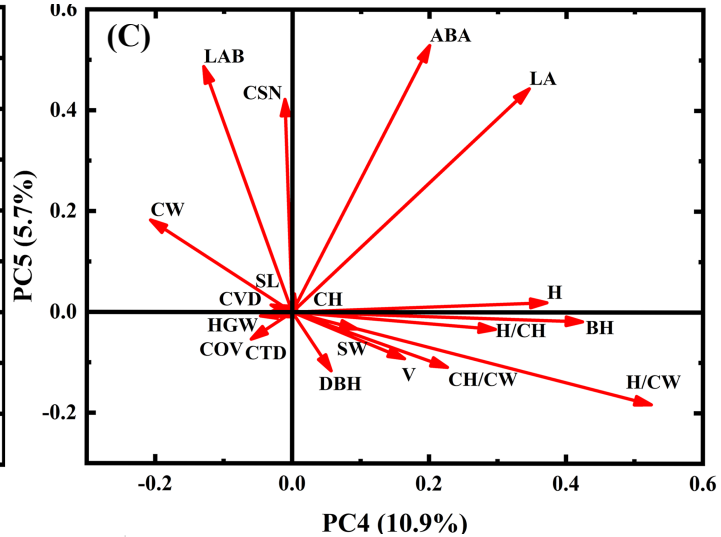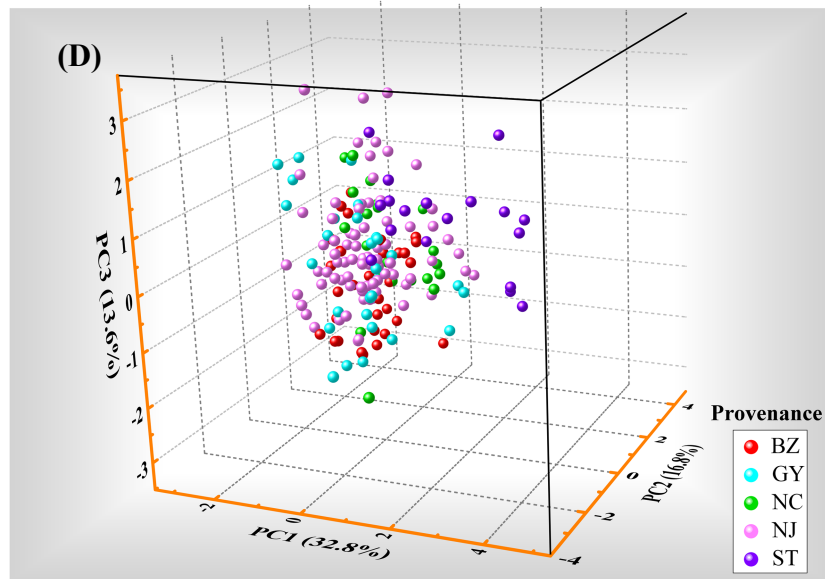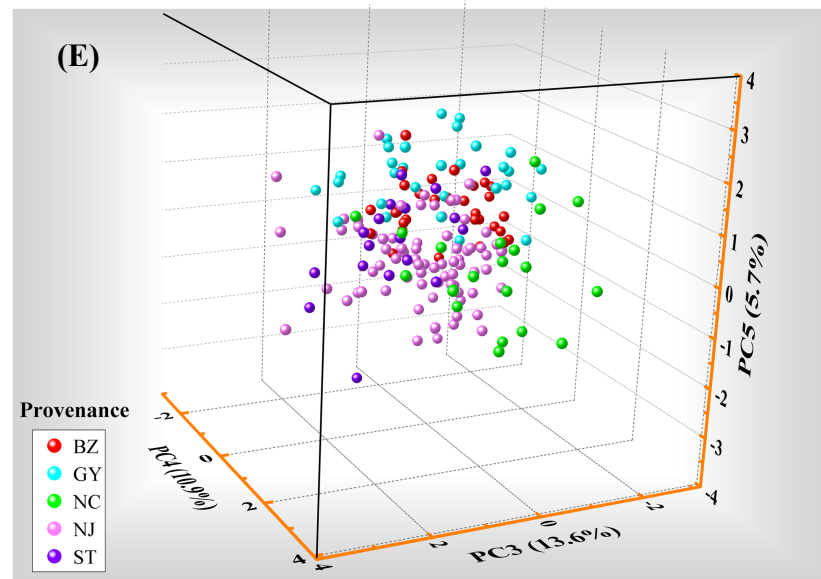

Supplement: Supplemental Information 20 — The projection of the load of 19 traits of Cupressus funebris on PC1 and PC2 (A), PC3 and PC4 (B), PC4 and PC5 (C), respectively. Figure 6 (D and E) shows the positions of 180 samples in three-dimensional space, with the coordinate axes being (D) PC1, PC2, and PC3, and (E) PC3, PC4, and PC5, respectively. Dfferent colors represent different provenances of Cupressus funebris, and the numbers on the axis are the eigenvalues of the five principal components. The information corresponding to the abbreviations can be found in Figure S2. [file peerj-12-18494-s020.pdf]

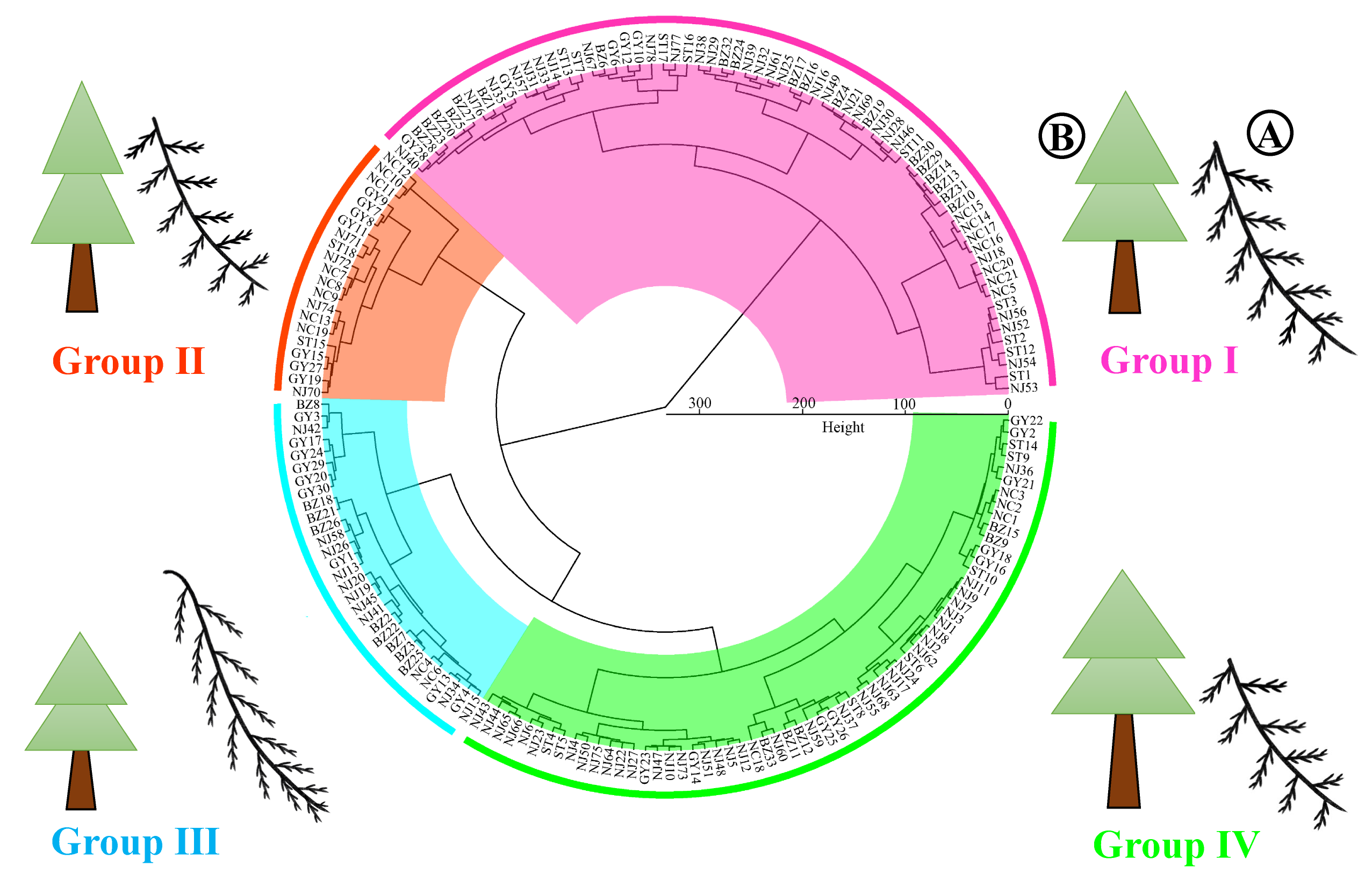

Supplement: Supplemental Information 21 — In the figure, different backgrounds and line colors represent different groups. (A) represents the branch morphology of Cupressus funebris. (B) represents the morphology of trees. [file peerj-12-18494-s021.png]

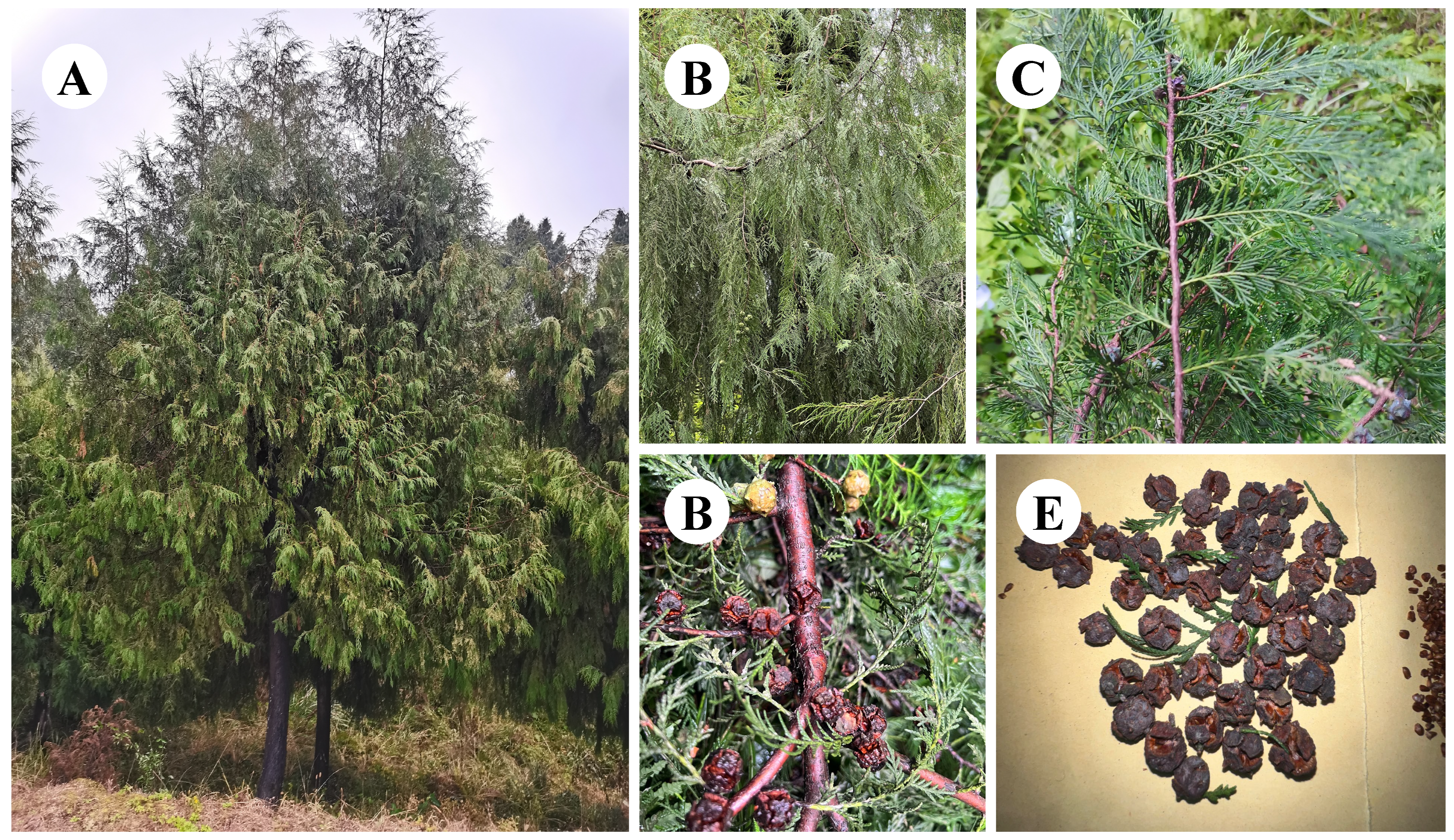

Supplement: Supplemental Information 22 — (A) juvenile Cupressus funebris tree morphology; (B) and (C) The morphology of Cupressus funebris branches and leaves; (D) and (E) The morphology of Cupressus funebris cones. [file peerj-12-18494-s022.png]
